# Pruebas funcionales en esclerosis múltiple y su comparabilidad con los valores de administración autónoma: estudio piloto

**DOI:** 10.7705/biomedica.6873

**Published:** 2023-09-30

**Authors:** Laura Estefanía Arenas-Vargas, Lorena López-Reyes, Simón Cárdenas-Robledo

**Affiliations:** 1 Centro de Esclerosis Múltiple, Hospital Universitario Nacional de Colombia, Bogotá, D.C., Colombia Hospital Universitario Nacional de Colombia Bogotá, D.C. Colombia; 2 Departamento de Medicina Interna, Facultad de Medicina, Universidad Nacional de Colombia, Bogotá, D.C., Colombia Universidad Nacional de Colombia Universidad Nacional de Colombia Bogotá, D.C. Colombia

**Keywords:** Esclerosis múltiple, telemonitorización, autoevaluación, evaluación de la discapacidad, valores de referencia, Multiple sclerosis, telemonitoring, self-testing, disability evaluation, reference values

## Abstract

**Introducción.:**

El deterioro neurológico en la esclerosis múltiple es variable para cada paciente y su cuantificación se dificulta con el tiempo. El *Multiple Sclerosis Outcome Assessment Consortium* estableció medidas clínicas sensibles, costo-efectivas y reproducibles para medir los resultados de los estudios clínicos. Sin embargo, sus valores de referencia se desconocen y, en la atención habitual, su uso no está extendido por limitaciones de tiempo y entrenamiento.

**Objetivo.:**

Establecer la factibilidad de la administración autónoma de las pruebas de marcha de 25 pies, símbolos y dígitos, y clavijas y nueve hoyos en individuos sanos.

**Materiales y métodos.:**

Se realizó un estudio piloto descriptivo. Se incluyeron individuos sanos entre los 18 y los 80 años. Las pruebas de *Timed 25-Foot Walking Test* (T25-FWT) [caminata cronometrada de 25 pies], *Symbol Digit Modality Test* (SDMT) [símbolos y dígitos] y *Nine-Hole Peg Test* (9-HPT) [clavijas y nueve agujeros] fueron administradas por un médico capacitado, quien también instruyó a los sujetos sobre la administración autónoma de las pruebas. La correlación y la concordancia entre la prueba guiada y la autónoma se evaluaron con los coeficientes de Pearson y Spearman, y el análisis gráfico de Bland-Altman.

**Resultados.:**

Se incluyeron 38 voluntarios sanos. La mediana de edad fue de 36 (rango: 23-55 años) y el 55,26 % eran mujeres. El coeficiente de correlación entre la prueba de administración guiada y la autónoma fue de 0,37 para la T25-FWT (p=0,01), de 0,54 para la SDMT (p<0,001) y de 0,64 y 0,65 para la 9-HPT, en las manos dominante y no dominante, respectivamente (p<0,001). Ambas formas de administración fueron concordantes para las pruebas T25-FWT (IC_95%_: -1,49 a 1,43), 9-HPT con la mano dominante (IC_95%_: -5,23 a 4,09), 9-HPT con la mano no dominante (IC_95%_: -7,75 a 7,14) y SDMT (IC_95%_: -20,94 a 24,10).

**Conclusiones.:**

Los resultados de este estudio ayudan a determinar los valores de normalidad poblacional obtenidos con las pruebas T25-FWT, 9-HPT y SDMT; además, establecen la posibilidad de practicarlas de forma autónoma.

## Abstract (en)

**Introduction.:**

Neurological impairment in multiple sclerosis is highly variable among patients and over time it is difficult to quantify. The Multiple Sclerosis Outcome Assessment Consortium established sensitive, cost-effective, clinically significant, and reproducible measures of different functional systems to measure outcomes in clinical trials. However, their use in clinical care routines is not widespread due to time and training constraints.

**Objective.:**

To evaluate the self-administration feasibility of the timed 25-foot walking, symbol-digit-modality, and 9-peg hole tests in healthy individuals.

**Materials and methods.:**

We performed a descriptive pilot study. Healthy individuals between 18 and 80 years of age were included. The Timed 25-Foot Walking Test (T25- FWT), the Symbol Digit Modality Test (SDMT), and the Nine-Hole Peg Test (9-HPT) (using the dominant and non-dominant hand) were administered by a trained physician, who also instructed the subjects about test self-administration. The correlation and agreement, between the guided and self-administered tests were assessed with Pearson and Spearman coefficients and the Bland-Altman method.

**Results.:**

Thirty-eight healthy volunteers were included. The median age was 36 (range: 23-55) years old, and 55.26% were female. The correlation coefficient between guided and selfadministered tests was 0.37 for the T25-FWT (p=0.01), 0.54 for the SDMT (p<0.001), and 0.64 and 0.65 for the 9-HPT, in the dominant and non-dominant hands, respectively (p<0,001). Both forms of administration were concordant for the T25-FWT (95%CI: -1,49 to 1,43), the 9-HPT with dominant hand (95%CI: -5,23 to 4,09), the 9-HPT with non-dominant hand (95%CI: -7,75 to 7,14) and the SDMT (95% CI: -20,94 to 24,10).

**Conclusions.:**

We provide a proof of concept related to the feasibility of the selfadministration of the T25-FWT, the 9-HPT, and the SDMT, as a tool to improve monitoring in routine clinical practice.

La esclerosis múltiple es una de las principales causas de discapacidad motora y cognitiva en personas jóvenes ^1^^,^^2^. Se estima que en el mundo hay cerca de tres millones de personas con esclerosis múltiple, con una prevalencia global de 35,9 casos por cada 100.000 habitantes ^3^. En Colombia, la prevalencia de la enfermedad se ha estimado en 7,5 casos por 100.000 habitantes y, en Bogotá, asciende a 16 casos por cada millón de habitantes ^4^.

La historia natural de la esclerosis múltiple es muy variable, pero se reconoce que la mayoría de los pacientes tienen una fase inicial de recurrencias y remisiones (esclerosis múltiple recurrente-remitente) ^5^. Una parte de estos pacientes tiende a presentar discapacidad progresiva tras este periodo (denominada esclerosis múltiple secundaria progresiva) y, en una minoría, la enfermedad se presenta de esta manera desde el principio (esclerosis múltiple primaria progresiva) ^6^. En años recientes, se ha encontrado que incluso pacientes con esclerosis múltiple remitente- recurrente tienden a presentar discapacidad en los periodos de remisión ^7^.

La esclerosis múltiple produce discapacidad en diferentes sistemas funcionales como la visión, la cognición, la destreza manual y la capacidad de marcha. Estas manifestaciones son muy variables entre pacientes y en un mismo paciente a lo largo del tiempo, lo que dificulta la cuantificación del estado de la discapacidad en un momento dado y de forma longitudinal.

La medida de discapacidad más utilizada en la práctica diaria y en estudios clínicos es la escala ampliada del estado de discapacidad (*Expanded Disability Status Scale*, EDSS) ^8^, que evalúa varios sistemas funcionales y la capacidad de marcha. Con esta escala, se puntúa a cada paciente entre 0 (examen neurológico normal) y 10 (muerte por esclerosis múltiple), con diferentes hitos intermedios, como la necesidad de apoyo unilateral (EDSS 6.0) o bilateral (EDSS 6.5) para la marcha, y la dependencia de la silla de ruedas para la movilidad (EDSS 7.0).

Esta escala tiene varias limitaciones reconocidas; las dos principales son que en etapas iniciales de la enfermedad, su sensibilidad para detectar progresión de la discapacidad es muy baja, y que, en estadios avanzados de discapacidad, su valoración se centra en la capacidad de marcha, desatendiendo otras funciones importantes como la cognitiva y la movilidad de los miembros superiores ^9^.

Por tales motivos, el *Multiple Sclerosis Outcome Assessment Consortium* estableció herramientas sensibles, prácticas, costo-efectivas, clínicamente significativas y reproducibles para medir los resultados de discapacidad cognitiva, visual, de marcha y de destreza manual en los estudios clínicos ^10^. Estas son el *Symbol Digit Modality Test* (SDMT) [símbolos y dígitos] ^11^, el *Timed 25-Foot Walking Test* (T25-FWT) [caminata cronometrada de 25 pies] ^12^, y *Nine-Hole Peg Test* (9-HPT) [clavijas y nueve agujeros] ^13^ y las pruebas de agudeza visual al contraste ^14^.

Sin embargo, se desconocen los valores normales de la población colombiana, razón por la cual no se cuenta con un parámetro para comparar los resultados de la valoración de un paciente. Además, dado que la administración de dichas pruebas requiere de equipos específicos y tiempo, no son ampliamente utilizadas en la práctica clínica habitual. Por lo tanto, es útil determinar si estas pruebas pueden ser practicadas de forma ambulatoria por el propio paciente.

Este estudio piloto tiene dos objetivos: proporcionar los datos necesarios para llevar a cabo un estudio ulterior para determinar los valores normales en nuestra población y evaluar la factibilidad de la aplicación autónoma de las pruebas de evaluación de resultados en esclerosis múltiple por parte de los pacientes afectados.

## Materiales y métodos

Este es un estudio piloto observacional de corte transversal, realizado en el Hospital Universitario Nacional de Colombia durante el año 2020. Los participantes fueron voluntarios de ambos sexos, entre los 18 y 80 años, sin manifestaciones clínicas neurológicas.

El muestreo se hizo por conveniencia. Para su inclusión en el estudio, los sujetos fueron sometidos a un examen neurológico que incluyó la evaluación de las funciones cognitivas (nivel de conciencia, orientación, atención, lenguaje y pensamiento), los nervios craneales, y la función motora y sensitiva. Se excluyeron los participantes con alteraciones de sus funciones cognitivas, motoras o sensitivas, y aquellos con una agudeza visual corregida menor de 20/50.

### Procedimientos y variables de estudio

Después de obtener el consentimiento para la participación en el estudio, se recolectaron los datos de las variables demográficas (edad, sexo, escolaridad, peso y talla) y un neurólogo capacitado en la administración de las pruebas de evaluación de resultados en esclerosis múltiple practicó las de marcha, destreza manual y cognitiva en los participantes.

**Figura 1:**
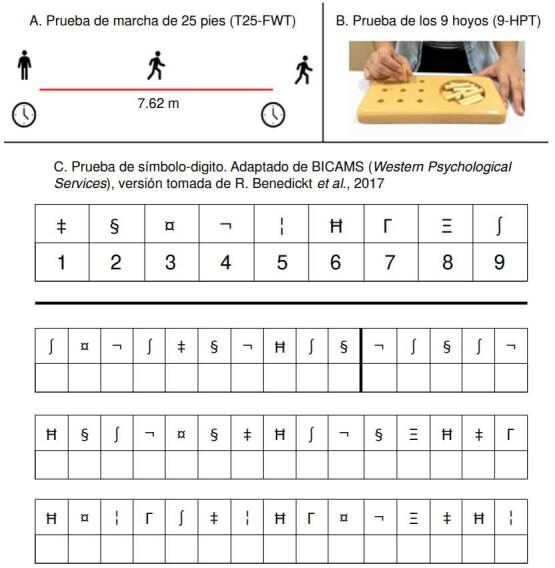
Pruebas de marcha (A), destreza manual (B) y velocidad de procesamiento (C) establecidas por el *Multiple Sclerosis Outcome Assessment Consortium* ^(8-11)^.

La prueba T25-FWT consiste en medir el tiempo que toma el paciente en caminar una distancia de 25 pies (7,62 m) (figura 1). Con la prueba 9-HPT, se mide el tiempo que le toma al paciente colocar nueve clavijas en sus hoyos respectivos y volver a retirarlos, con una sola mano, en un dispositivo de madera (figura 1B). La unidad de medida de estas dos pruebas es el tiempo aproximado a décimas de segundo del promedio de dos evaluaciones. En el caso de la prueba 9-HPT, se examinan de forma independiente la mano dominante y la no dominante. Los sujetos ambidextros fueron analizados como diestros.

La prueba SDMT consiste en asignar números del 1 al 9 a diferentes símbolos, según una clave establecida (figura 1C). Se puntúa contando el número de símbolos que se asignaron correctamente en un lapso de 90 segundos; el reporte puede hacerse de forma verbal o escrita. Dado el potencial compromiso de la destreza manual o visual en las personas con esclerosis múltiple ^15^, se decidió evaluar el reporte verbal.

La muestra de participantes se dividió en dos grupos. En uno de ellos, se administraron las pruebas y, luego, se entregó una carta de instrucciones a los participantes para que las aplicaran de forma autónoma. En el segundo grupo, se llevó a cabo el mismo procedimiento, pero en orden inverso. Para este estudio se omitieron las pruebas de agudeza y contraste visual, porque su administración requiere de implementos específicos (cartillas de Sloan y Pelli-Robson), además de una iluminación estándar y la guía por parte de personal entrenado. Dado que la mayoría de las instituciones no cuentan con estas facilidades, la administración por parte del paciente no es viable.

### Tamaño de muestra y análisis estadístico

La muestra se calculó con una técnica no probabilística de muestreo. Teniendo en cuenta un poder del 90 %, un valor alfa de 0,01 y un valor esperado de 3,0 ± 1,5 s, se calculó un tamaño de muestra de 37 participantes para la T25-FWT. Ya que se desconocen completamente los valores de referencia en la población colombiana, se tomaron una media y una desviación estándar arbitraria para el resultado esperado de la T25-FWT.

Las variables cuantitativas se describieron con medidas de tendencia central (medias, medianas) y dispersión (desviación estándar: DE; rangos intercuartílicos; Q_1_-Q_3_) de acuerdo con su distribución estadística. Las variables cualitativas se expresaron en términos de frecuencias absolutas y relativas.

Dado que el desempeño del participante en cada prueba puede afectarse por diferentes variables, se categorizó la muestra en tres rangos de edad (18 a 30 años, 31 a 50 años y más de 50 años), los cuales fueron analizados luego con la prueba de Kruskal-Wallis.

Se evaluó la correlación entre las mediciones obtenidas de las pruebas por administración guiada y las de las practicadas de forma autónoma, mediante los coeficientes de Pearson o Spearman, según la distribución de los datos.

**Cuadro 1:** Distribución de variables demográficas (n=38)

Sexo, n (%)	Femenino	21	(55,26)
	Masculino	17	(44,74)
Escolaridad, n (%)	Primaria y secundaria	11	(28,95)
	Técnica	7	(18,42)
	Universitaria y postgrado	20	(52,63)
Lateralidad, n (%)	Diestra	29	(76,32)
	Zurda	6	(15,79)
	Ambidextra	3	(7,89)
Edad (años), mediana (Q1-Q3)		36	(23-55)
Talla (m), media (DE)		1,65	(0,07)
Peso (kg), media (DE)		66,83	(11,45)
IMC, mediana (Q1-Q3)		22,83	(21,30 - 26,34)

Q_1_-Q_3_: rango intercuartílico; DE: desviación estándar; IMC: índice de masa corporal

La concordancia entre la administración guiada y la autónoma se evaluó con el método de Bland-Altman. Este método es una medida absoluta de concordancia, que permite determinar la variabilidad o precisión de dos mediciones (en este caso, la administración guiada y la autónoma), establecer los límites de concordancia y definir si las mediciones son concordantes en el rango de valores medidos. Consiste en el cálculo de la diferencia de las observaciones de los dos métodos frente a la media de las observaciones de las técnicas en comparación y la desviación típica de las diferencias.

Por último, se calcularon los límites de concordancia, que corresponden a la diferencia media más o menos dos veces la desviación estándar. Cuando el intervalo de las diferencias de las medias incluye el cero, se puede considerar que las dos mediciones son concordantes ^16^^,^^17^.

Los datos se recolectaron por medio de la plataforma REDcap ^18^ y se analizaron con Stata, versión 15.0.

### Consideraciones éticas y disponibilidad de los datos

Este estudio fue aprobado por el Comité de Ética en Investigación del Hospital Universitario Nacional de Colombia. Los datos anonimizados se encuentran disponibles, previa solicitud razonable al autor de correspondencia y la autorización por dicho Comité.

## Resultados

Se evaluaron 38 sujetos (17 hombres y 21 mujeres). En el cuadro 1 se resumen las variables sociodemográficas de la muestra evaluada.

Los participantes realizaron las pruebas por administración guiada y autónoma. Para la administración guiada, la mediana de la prueba T25-FW fue de 3,97 s (Q_1_-Q_3_ = 3,68-4,40). La media para la prueba 9-HPT para la mano dominante fue de 19,68 (DE = 2,88) s y, para la mano no dominante, la mediana fue de 21,70 s (Q_1_-Q_3_ = 17,96-23,27). La mediana de la prueba SDMT fue de 64,60 (Q_1_-Q_3_ = 59-70) identificaciones correctas. Los resultados de las formas de administración autónoma y de la guiada se muestran en el cuadro 2.

**Cuadro 2:** Resultados de las pruebas según la forma de administración

Prueba	Mediana (Q1-Q3) o media (DE)	Mínimo	Máximo	Correlación	p
T25-FW-a	3,97 (3,60 - 4,40)	3,05	5,88	0,37 ^a^	0,01
T25-FW-g	3,97 (3,68 - 4,40)	3,15	5,85		
9-HPT-a con mano dominante	19,68 (2,88)	14,53	25,65	0,64 ^b^	<0,001
9-HPT-g con mano dominante	20,28 (2,71)	14,53	25,34		
9-HPT-a con mano no dominante	21,59 (18,05 - 23,49)	14,93	25,50	0,65 ^a^	<0,001
9-HPT-g con mano no dominante	21,70 (17,96 - 23,27)	11,79	39,78		
SDMT-a	62,50 (60 - 71)	50	85	0,54 ^a^	<0,001
SDMT-g	64,60 (59 - 70)	24	90		

^a^ Coeficiente de correlación de Spearman; ^b^ Coeficiente de correlación de Pearson; Q_1_-Q_3_: rango intercuartílico; DE: desviación estándar; T25-FW-a: prueba de 25 pies autoadministrada; T25-FW-g: prueba de 25 pies, guiada; 9-HPT-a: prueba de 9 hoyos, autoadministrada; 9-HPT-g: prueba de 9 hoyos, guiada; SDMT-a: prueba de dígito-símbolo, autoadministrada; SDMT-g: prueba de dígito-símbolo, guiada

**Figura 2:**
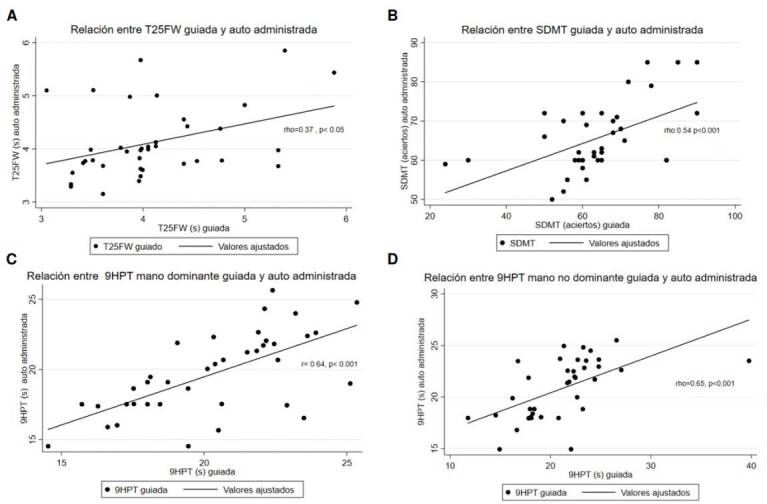
Correlación entre las formas de administración para las pruebas (A) T-25FW, (B) símbolos y dígitos, (C) 9-HPT con mano dominante y (D) 9-HPT con mano no dominante

Según la evaluación de la normalidad de la distribución de los datos, se utilizó el coeficiente de correlación de Pearson para la 9-HPT con la mano dominante y, para el resto de las pruebas, se utilizó el coeficiente de correlación de Spearman. La correlación entre ambas formas de administración de las tres pruebas fue significativa, con un coeficiente de correlación de 0,37 para la T25-FW (p = 0,01), de 0,54 para la SDMT (p < 0,001), y 0,64 y 0,65 para la 9-HPT en la manos dominante y la no dominante, respectivamente (p < 0,001 para ambas pruebas) (cuadro 2 y figura 2). El test de Kruskal-Wallis mostró diferencias significativas (p < 0,05) por grupos de edad para la T25-FW guiada, la 9-HPT con mano dominante, autoadministrada y guiada, y para la 9-HPT con mano no dominante autoadministrada (cuadro 3).

**Cuadro 3:** Comparación de las medianas (Q1-Q3) entre las dos formas de administración, según los rangos de edad

	18-30 años (n=16)	31-50 años (n=9)	> 50 años (n=13)	Test de Kruskal-Wallis (p)
T25-FW-a	3,85 (3,42 - 4,06)	3,98 (3,96 - 4,4)	4,13 (3,97 - 4,77)	0,1
T25-FW-g	3,79 (3,51 - 4,01)	3,99 (3,82 - 4,42)	4,04 (3,78 - 5,19)	0,01
9-HPT-a con mano dominante	17,53 (16,64 - 19,10)	21,23 (20,39 - 21,72)	21,89 (18,65 - 22,62)	0,01
9-HPT-g con mano dominante	18,59 (17,07 - 20,41)	21,84 (20,67 - 22,07)	22,17 (19,06 - 23,61)	0,01
9-HPT-a con mano no dominante	18,60 (17,96 - 21,62)	22,49 (21,47 - 23,53)	22,82 (18,83 - 23,63)	0,02
9-HPT-g con mano no dominante	19,93 (18,00 - 22,27)	21,86 (20,95 - 22,39)	23,37 (17,96 - 24,81)	0,26
SDMT-a	65 (61 - 72)	61 (60 - 65)	66 (60 - 70)	0,37
SDMT-g	65 (61 - 80)	63 (59 - 70)	59 (52 - 80)	0,07

Q_1_-Q_3_: rango intercuartílico; T25-FW-a: prueba de caminata cronometrada de 25 pies, autoadministrada; T25-FW-g: prueba de caminata cronometrada de 25 pies, guiada; 9-HPT-a: prueba de clavijas y nueve agujeros, autoadministrada; 9-HPT-g: prueba de clavijas y nueve agujeros, guiada; SDMT-a: prueba de símbolos y dígitos, autoadministrada; SDMT-g: prueba de símbolos y dígitos, guiada

**Figura 3:**
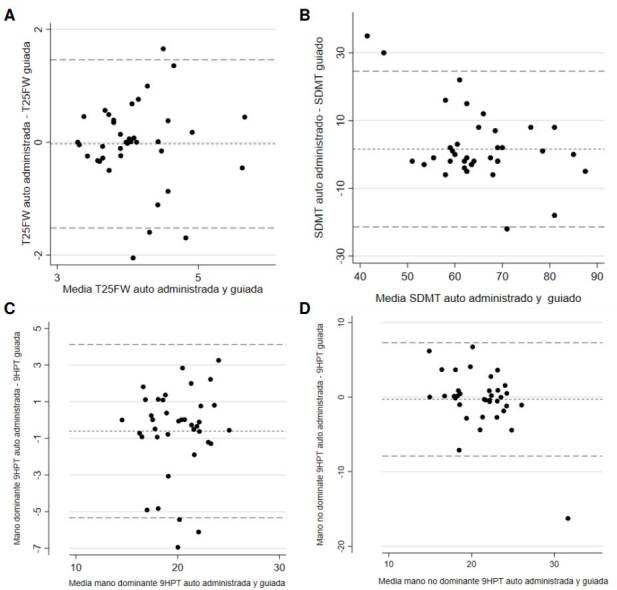
Análisis de Bland-Altman para la evaluación de concordancia entre la administración dirigida y la autoadministración de las pruebas: (A) T25-FW (B) SDMT, (C) 9-HPT con mano dominante y (D) 9-HPT con mano no dominante. Las líneas punteadas representan los límites superior e inferior del intervalo de confianza del 95 %.

Se encontró una concordancia significativa entre las dos formas de administración. Para la T25-FW, la media de las diferencias entre la administración guiada y la autónoma, fue de -0,02 s (IC_95%_: -1,49 a 1,43; DE = 0,74). Para la 9-HPT con la mano dominante, la media de las diferencias entre la administración guiada y la autónoma fue de -0,69 s (IC_95%_: -5,23 a 4,09; DE: 2,36), mientras que para la mano no dominante, la media de las diferencias entre la administración guiada y la autónoma fue de -0,30 s (IC_95%_: -7,75 a 7,14; DE = 3,80). Finalmente, para la SDMT, la media de las diferencias entre la administración guiada y la autónoma fue de 1,57 respuestas correctas (IC_95%_: -20,94 a 24,10; DE = 11,49) (figura 3).

## Discusión

Este estudio piloto determina las medias o medianas de las pruebas T25-FW, 9-HPT y SDMT, según la edad y establece la posibilidad de su administración autónoma en personas sanas.

Desde la publicación inicial de la prueba SDMT ^19^, se han llevado a cabo estudios en búsqueda de valores normales para diferentes poblaciones con resultados para la versión escrita ^20^^-^^23^ y la verbal ^24^^,^^25^. Las medias encontradas en estos estudios son bastante divergentes, con 51,3 ^24^ y 32,4 ^25^ respuestas correctas. Ambas difieren de la mediana de 64,6 (Q_1_-Q_3_ = 59-70) encontrada en este estudio, lo que resalta la importancia de determinar valores normales de cada población. La mayoría de los datos de estas publicaciones, al igual que en el presente trabajo, coinciden en que existen diferencias significativas según la edad y el nivel educativo ^24^^,^^25^. Por lo tanto, es importante tener en cuenta estas variables para estratificar la muestra en estudios futuros.

Con respecto a las pruebas T25-FW y 9-HPT, la información de valores normativos es más escasa. Se ha evaluado la T25-FW en grandes cohortes de pacientes con esclerosis múltiple, reportándose una media de 6,8 s (DE = 3,1) ^26^. Sin embargo, se desconocen los valores de esta prueba en poblaciones sanas. Para la 9-HPT, Mathiowetz *et al*^27^ describieron valores normales con resultados muy similares a los de este estudio: media de hombres con mano derecha igual a 19,0 s (DE = 3,2) y con mano izquierda igual a 20,6 s (DE = 3,9); y mujeres con mano derecha igual a 17,9 s (DE = 2,8) y con mano izquierda igual a 19,6 s (DE = 3,4), en una muestra de 618 voluntarios sanos. Resultados similares se documentaron en Bangladesh, aunque con una muestra menor (n=180) ^28^.

Si bien en el presente estudio no fue posible determinar los valores normales por ausencia de un valor de referencia para calcular un adecuado tamaño muestral, los datos obtenidos permitirán calcular la muestra requerida y obtener el poder estadístico necesario para establecer valores de normalidad en la población colombiana.

Además de informar la desviación de la función de un paciente en comparación con los valores normales de su población, las pruebas del *Multiple Sclerosis Outcome Assessment Consortium* permiten cuantificar el cambio en la función de forma longitudinal. En ese sentido, se considera que el cambio, al menos, del 20 % entre una medición y otra, en un mismo paciente, representa significancia clínica ^26^ y predicen el incremento del estado de discapacidad a largo plazo, independientemente del puntaje basal ^29^.

Así, si un paciente tiene una reserva cognitiva alta y un puntaje muy superior al normativo en la SDMT, en el momento del diagnóstico o del inicio del tratamiento, es posible que, en el transcurso de un tiempo determinado, la enfermedad genere un detrimento que interfiera en el desarrollo de sus funciones diarias. Sin embargo, puede que este detrimento no afecte tanto el puntaje como para considerado anormal al compararlo con el valor normativo de la población sana. Esto es importante porque permite detectar cambios tempranos en la función neurológica, antes de cambios evidenciables en el puntaje del estado de discapacidad, y tomar decisiones terapéuticas más oportunas.

La correlación entre las dos formas de administración fue débil para la T25-FW (ρ = 0,37), media para la SDMT (ρ = 0,54) y fuerte para la 9-HPT (ρ = 0,61 con mano dominante y 0,65 con mano no dominante), pero fue significativa en todos los casos. Dado que la variabilidad entre ambas formas de administración es similar según lo encontrado con el método de Bland- Altman, se sugiere la aplicación autónoma de las pruebas.

Los estudios en que han evaluado la correlación de estas pruebas en diferentes formas de aplicación (guiada o autónoma) en personas con esclerosis múltiple, han contribuido con sus resultados al desarrollo de herramientas digitales que permiten la evaluación funcional autónoma de los pacientes. La tecnología portátil permite que las personas lleven consigo sensores para un monitoreo continuo y remoto de las enfermedades.

En la esclerosis múltiple, se han evaluado productos para medir aspectos como marcha, cognición, actividad, estado de ánimo y fatiga, principalmente por medio de aplicaciones para teléfonos inteligentes, considerando costo, adaptabilidad del paciente y precisión ^30^^-^^32^. Lam *et al*. investigaron la utilidad de la SDMT adaptada a teléfonos inteligentes para detectar y monitorear la disfunción cognitiva en personas con esclerosis múltiple. Evaluaron la confiabilidad con coeficientes de correlación intraclase, la validez del constructo (análisis entre deterioro cognitivo, la conservación cognitiva y los controles sanos), y la validez concurrente (coeficientes de correlación). Los resultados mostraron confiabilidad (alta correlación test-retest) y capacidad para detectar diferencias entre pacientes con deterioro cognitivo, conservación cognitiva y personas sanas ^33^.

También, se desarrolló la “prueba de velocidad de procesamiento”, una herramienta en el iPad para medir la disfunción cognitiva en pacientes con esclerosis múltiple. Esta prueba mostró confiabilidad, correlación con la SDMT, sensibilidad para discriminar entre pacientes y controles sanos, y utilidad para detectar déficits de velocidad de procesamiento en la esclerosis múltiple ^34^.

Con la aplicación “dreaMS”y mediante 11 pruebas, se evalúan la marcha, el equilibrio, la destreza manual, la cognición y la visión. Se evaluó su confiabilidad con coeficientes de correlación intraclase y coeficientes de variación mediana, y se consultó a los participantes con esclerosis múltiple sobre la significación y la aceptación de las pruebas. De las 133 características extraídas, 89 cumplieron con los criterios de confiabilidad.

Todas las pruebas se consideraron significativas y útiles por las personas con esclerosis múltiple. Además, la observancia de la aplicación fue alta, con un promedio del 96 % de las pruebas realizadas en los horarios programados.

En el estudio se concluyó que “dreaMS” es confiable y significativa para medir funciones neurológicas en personas con esclerosis múltiple ^35^. Se destacó la importancia de estudios a más largo plazo para validar estas medidas digitales y su potencial como biomarcadores en el monitoreo de la esclerosis múltiple. En caso de comprobarse su viabilidad, la administración autónoma de las pruebas del *Multiple Sclerosis Outcome Assessment Consortium*, por parte de las personas con esclerosis múltiple será una herramienta adicional para el seguimiento clínico, con medidas validadas y extensamente probadas en estos pacientes.

Este estudio tiene ciertas limitaciones que deben tenerse en cuenta. La más relevante es el sesgo de selección derivado de la técnica de muestreo, porque los participantes pueden no ser una muestra representativa de la población general en términos de distribución de variables demográficas. Por lo tanto, para determinar los valores normales deben tenerse en cuenta estas variables en un muestreo estratificado, con el fin de tener una muestra representativa.

Además, el diseño del estudio permitió que los participantes realizaran las pruebas de forma autónoma, sin un control estricto de las condiciones de su aplicación. Esto podría introducir sesgo de medición, pero, en parte, contribuye a que los resultados sean aplicables en las circunstancias en que se pretende implementar en las personas con esclerosis múltiple: la administración en casa sin supervisión.

Este estudio no involucró pacientes con esclerosis múltiple, que es la población objetivo de la administración autónoma de las pruebas. Es posible que los resultados obtenidos en voluntarios sanos no sean extrapolables a los pacientes, razón por la cual se requiere su confirmación en personas con esclerosis múltiple. Sin embargo, se considera que de no ser viable la administración autónoma de estas pruebas en personas sin déficit cognitivo o motor, tampoco lo será en personas con una enfermedad neurológica como la esclerosis múltiple.

Finalmente, en el estudio no se evaluaron los cambios longitudinales en los resultados de las pruebas, lo cual es deseable para el seguimiento a largo plazo de los pacientes.

Los resultados de este estudio piloto dan paso a la determinación de valores de normalidad poblacional de las pruebas T25-FW, SDMT y 9-HPT en estudios futuros, y establecen la posibilidad de su administración autónoma, previa confirmación con estudios en pacientes con esclerosis múltiple. De hacerlo, se podría contar con herramientas con validez clínica y estadística para la evaluación prospectiva y autónoma de las personas con esclerosis múltiple.
